# 11-(2-Oxopyrrolidin-1-ylmeth­yl)-1,2,3,4,5,6,11,11a-octa­hydro­pyrido[2,1-*b*]quinazolin-6-one dihydrate

**DOI:** 10.1107/S1600536810009955

**Published:** 2010-03-20

**Authors:** Zarif U. Samarov, Rasul Ya. Okmanov, Kambarali K. Turgunov, Bakhodir Tashkhodjaev, Khusnutdin M. Shakhidoyatov

**Affiliations:** a S.Yunusov Institute of the Chemistry of Plant Substances, Academy of Sciences of Uzbekistan, Mirzo Ulugbek Str. 77, Tashkent 100170, Uzbekistan

## Abstract

In the crystal structure of the title compound, C_17_H_21_N_3_O_2_·2H_2_O, water mol­ecules are mutually O—H⋯O hydrogen bonded and form infinite chains propagating along the *b* axis. Neighboring chains are linked by the quinazoline mol­ecules by means of O—H⋯O=C hydrogen bonds, forming a two–dimensional network.

## Related literature

For general background to pyrido-quinazoline alkaloids and their structures, see: Fitzgerald *et al.* (1966 ▶); Tashkhodzhaev *et al.* (1995 ▶); Turgunov *et al.* (2003 ▶); Tozhiboev *et al.* (2007 ▶). For the synthesis of pyrido-quinazolinone derivatives, see: Shakhidoyatov (1983 ▶); Barakat (1998 ▶). For chemical modifications of pyrido-quinazoline alkaloids, see: Shakhidoyatov *et al.* (2007 ▶). For the amido­methyl­ation reaction of quinazolinone derivatives, see: Pandey *et al.* (2008 ▶); Ibragimov *et al.* (2004 ▶). For bond-length data, see: Allen *et al.* (1987 ▶).
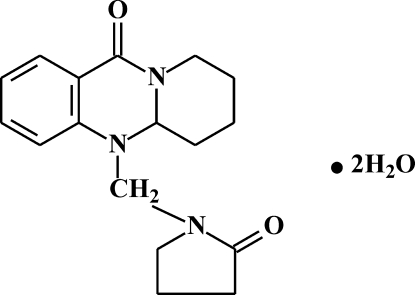


## Experimental

### 

#### Crystal data

C_17_H_21_N_3_O_2_·2H_2_O
*M* *_r_* = 335.40
Monoclinic, 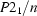

*a* = 14.794 (3) Å
*b* = 7.6720 (15) Å
*c* = 15.593 (3) Å
β = 104.48 (3)°
*V* = 1713.6 (6) Å^3^
*Z* = 4
Mo *K*α radiation
μ = 0.09 mm^−1^
*T* = 300 K
0.60 × 0.55 × 0.35 mm

#### Data collection

Stoe Stadi-4 four-circle diffractometer
3261 measured reflections
3005 independent reflections
2457 reflections with *I* > 2σ(*I*)
3 standard reflections every 60 min  intensity decay: 1.8%

#### Refinement

*R*[*F* ^2^ > 2σ(*F* ^2^)] = 0.045
*wR*(*F* ^2^) = 0.121
*S* = 1.10
3005 reflections
234 parameters
H atoms treated by a mixture of independent and constrained refinement
Δρ_max_ = 0.18 e Å^−3^
Δρ_min_ = −0.14 e Å^−3^

### 

Data collection: *STADI4* (Stoe & Cie, 1997 ▶); cell refinement: *STADI4*; data reduction: *X-RED* (Stoe & Cie, 1997 ▶); program(s) used to solve structure: *SHELXS97* (Sheldrick, 2008 ▶); program(s) used to refine structure: *SHELXL97* (Sheldrick, 2008 ▶); molecular graphics: *XP* (Bruker, 1998 ▶); software used to prepare material for publication: *SHELXL97*.

## Supplementary Material

Crystal structure: contains datablocks I, global. DOI: 10.1107/S1600536810009955/bq2200sup1.cif

Structure factors: contains datablocks I. DOI: 10.1107/S1600536810009955/bq2200Isup2.hkl

Additional supplementary materials:  crystallographic information; 3D view; checkCIF report

## Figures and Tables

**Table 1:** Hydrogen-bond geometry (Å, °)

*D*—H⋯*A*	*D*—H	H⋯*A*	*D*⋯*A*	*D*—H⋯*A*
Ow1—Hw1⋯O1	0.84 (4)	2.00 (4)	2.818 (3)	167 (3)
Ow1—Hw2⋯Ow2	0.88 (3)	1.83 (3)	2.703 (3)	172 (3)
Ow2—Hw4⋯Ow1^i^	0.86 (3)	1.88 (3)	2.733 (3)	177 (3)
Ow2—Hw3⋯O2^ii^	0.90 (3)	1.86 (3)	2.764 (3)	178 (3)

Symmetry codes: (i) 
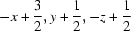
; (ii) 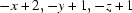.

**Fig. 1.**
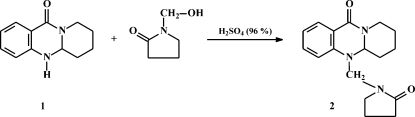
Reaction sequence for (I).

**Fig. 2.**
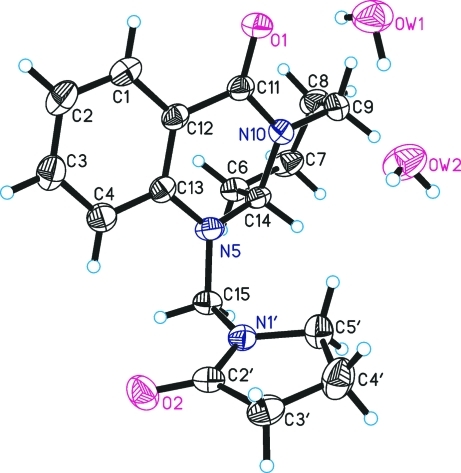
The asymmetric part of crystalline cell, showing 30% probability displacement ellipsoids and the atom-numbering scheme.

**Fig. 3.**
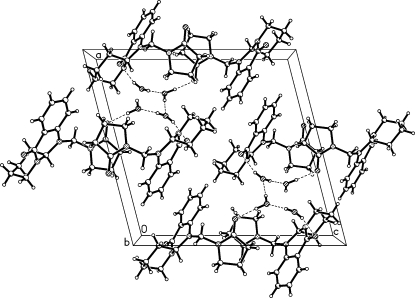
Packing view of the title compound and H-bonds networks in the crystal.

## supplementary crystallographic information

### Comment

Alkaloid pyrido-quinazoline derivatives are widespread compounds in plants (Fitzgerald *et al.*, 1966), was elaborated simple and convenient method of synthesis (Shakhidoyatov 1983; Barakat 1998), was studied structure and modification of pyrido-quinazoline derivatives (Tashkhodzhaev *et al.*, 1995; Turgunov *et al.*, 2003; Shakhidoyatov *et al.*, 2007; Tozhiboev *et al.*, 2007).

Amidomethylation (Pandey *et al.*, 2008; Ibragimov *et al.*, 2004) of 1,2–dihydro derivatives tricyclic quinazolin–4–ones allows to enter in molecule alkyl group and to get the series of the new compounds. For this purpose is realized amidomethylation of 5,6,7,8,9,14–hexahydropyrido[2,1–*d*]quinazolin–11–one with *N*–methylolpyrrolidin–2–one. Concentrated sulfuric acid has chosen as a catalyst and the reaction carried out at room temperature (Figure 1).

The molecular structure of the title compound is shown in Figure 2. Quinazoline ring (with exclusion of atom C14) and *N*-methylolpyrrolidin-2-one ring with inclusion of atom N5 are planar and angle between plans is 77.38 (6)°. Pyrimidine ring takes conformation of sofa leaving the atom C14 from the plane of rest five atoms on 0.409 Å. The third cycle, containing piperidine ring, has conformation of chair.

In the molecule the length of C11═O1 bond (1.241 (2) Å) noticeably, but C2'═O2 bond (1.228 (2) Å) slightly elongated from generally accepted value of C═O bond (Allen *et al.*, 1987). The elongation and planarity of valence bonds of atoms of N10 and N1' indicate conjugation of π-electronic system of carbonic group with not divided electronic pairs of corresponding nitrogen atoms, in case C11═O1 in conjugation participates additionally aromatic ring.

In asymmetric part of crystal cell there are two molecules of water and one molecule of quinazoline derivative (Figure 2). Molecules of water are connected by hydrogen bonds Ow1—H···Ow2 and Ow2—H···Ow1 and form the infinite chain along b-axis. These hydrogen bond chains are linked by hydrogen bonds of Ow1—H···O1═C10 and Ow2—H···O2═C2' forming two–dimensional network. Hydrogen bond parameters are shown in Table 1 and packing of molecules with hydrogen bonds are shown on Figure 3.

### Experimental

0.606 g (3 mmol) of the compound 1 is added to 1.8 ml concentrated sulfuric acid (96%) holding temperature below than 278 K. Then under mixing is added by portion 0.351 g (3 mmol) *N*-methylolpyrrolidone-2 during 2.5 hours. Reactionary mixture left on night, next day to reaction mixture is added ice and neutralized by ammonia. Precipitate of compound 2 is filtered, washed with water, dried and re-crystallized from hexane, yield 0.9 g (94%).

Colorless crystals, suitable for X-ray (in the form of the prisms and with size 0.60x0.55x0.35 mm) were grown from 1:1 mixture of aqueous methanol and tetrachloromethane at room temperature, mp. 373 K.

### Refinement

The hydrogen atoms of the water molecules were located from difference of Fourier synthesis, the O—H distances are between 0.84 (4) – 0.90 (3) Å. All other H atoms bonded to C atoms were placed geometrically (with C—H distances of 0.97 Å for CH_2_ and 0.93 Å for Car) and included in the refinement in riding motion approximation with Uĩso~=1.2U~eq~(C) [Uĩso~=1.5U~eq~(C) for methyl H atoms].

### Crystal data

**Table:**

C_17_H_21_N_3_O_2_·2H_2_O	*F*(000) = 720
*M**_r_* = 335.40	*D*_x_ = 1.300 Mg m^−^^3^
Monoclinic, *P*2_1_/*n*	Melting point: 373(1) K
Hall symbol: -P 2yn	Mo *K*α radiation, λ = 0.71073 Å
*a* = 14.794 (3) Å	Cell parameters from 15 reflections
*b* = 7.6720 (15) Å	θ = 10–20°
*c* = 15.593 (3) Å	µ = 0.09 mm^−^^1^
β = 104.48 (3)°	*T* = 300 K
*V* = 1713.6 (6) Å^3^	Prizm, yellow
*Z* = 4	0.60 × 0.55 × 0.35 mm

### Data collection

**Table:**

Stoe Stadi-4 four-circle diffractometer	*R*_int_ = 0.0000
Radiation source: fine-focus sealed tube	θ_max_ = 25.0°, θ_min_ = 1.7°
graphite	*h* = −17→17
Scan width (ω) = 1.56 – 1.68, scan ratio 2θ:ω = 1.00 I(Net) and σ(I) calculated according to Blessing (1987)	*k* = 0→9
3261 measured reflections	*l* = 0→18
3009 independent reflections	3 standard reflections every 60 min
2457 reflections with *I* > 2σ(*I*)	intensity decay: 1.8%

### Refinement

**Table:**

Refinement on *F*^2^	Secondary atom site location: difference Fourier map
Least-squares matrix: full	Hydrogen site location: inferred from neighbouring sites
*R*[*F*^2^ > 2σ(*F*^2^)] = 0.045	H atoms treated by a mixture of independent and constrained refinement
*wR*(*F*^2^) = 0.121	*w* = 1/[σ^2^(*F*_o_^2^) + (0.0469*P*)^2^ + 0.6922*P*] where *P* = (*F*_o_^2^ + 2*F*_c_^2^)/3
*S* = 1.10	(Δ/σ)_max_ < 0.001
3005 reflections	Δρ_max_ = 0.18 e Å^−^^3^
234 parameters	Δρ_min_ = −0.14 e Å^−^^3^
0 restraints	Extinction correction: *SHELXL97* (Sheldrick, 2008), Fc^*^=kFc[1+0.001xFc^2^λ^3^/sin(2θ)]^-1/4^
Primary atom site location: structure-invariant direct methods	Extinction coefficient: 0.0101 (13)

### Special details

**Table:**

Geometry. All esds (except the esd in the dihedral angle between two l.s. planes) are estimated using the full covariance matrix. The cell esds are taken into account individually in the estimation of esds in distances, angles and torsion angles; correlations between esds in cell parameters are only used when they are defined by crystal symmetry. An approximate (isotropic) treatment of cell esds is used for estimating esds involving l.s. planes.
Refinement. Refinement of *F*^2^ against ALL reflections. The weighted *R*-factor wR and goodness of fit *S* are based on *F*^2^, conventional *R*-factors *R* are based on *F*, with *F* set to zero for negative *F*^2^. The threshold expression of *F*^2^ > σ(*F*^2^) is used only for calculating *R*-factors(gt) etc. and is not relevant to the choice of reflections for refinement. *R*-factors based on *F*^2^ are statistically about twice as large as those based on *F*, and *R*- factors based on ALL data will be even larger.

### Fractional atomic coordinates and isotropic or equivalent isotropic displacement parameters (Å^2^)

**Table:**

	*x*	*y*	*z*	*U*_iso_*/*U*_eq_	
O1	0.96440 (10)	0.22400 (19)	0.12560 (11)	0.0653 (4)	
O2	1.12720 (11)	0.8019 (2)	0.50954 (9)	0.0681 (5)	
C1	1.14106 (15)	0.2498 (3)	0.24497 (14)	0.0570 (5)	
H1A	1.1289	0.1515	0.2091	0.068*	
C2	1.22228 (16)	0.2586 (3)	0.31069 (15)	0.0661 (6)	
H2A	1.2656	0.1684	0.3187	0.079*	
C3	1.23851 (15)	0.4034 (3)	0.36460 (14)	0.0613 (6)	
H3A	1.2927	0.4090	0.4102	0.074*	
C4	1.17602 (14)	0.5405 (3)	0.35233 (13)	0.0516 (5)	
H4A	1.1880	0.6363	0.3901	0.062*	
N5	1.03125 (11)	0.6705 (2)	0.26631 (10)	0.0474 (4)	
C6	0.98631 (14)	0.7776 (3)	0.10982 (13)	0.0512 (5)	
H6A	1.0385	0.7208	0.0940	0.061*	
H6B	1.0053	0.8949	0.1297	0.061*	
C7	0.90303 (17)	0.7853 (3)	0.02918 (14)	0.0649 (6)	
H7A	0.8542	0.8560	0.0431	0.078*	
H7B	0.9220	0.8403	−0.0196	0.078*	
C8	0.86498 (16)	0.6055 (3)	0.00118 (14)	0.0681 (7)	
H8A	0.9097	0.5419	−0.0230	0.082*	
H8B	0.8077	0.6163	−0.0451	0.082*	
C9	0.84574 (14)	0.5042 (3)	0.07796 (16)	0.0648 (6)	
H9A	0.7944	0.5577	0.0966	0.078*	
H9B	0.8280	0.3857	0.0594	0.078*	
N10	0.92898 (11)	0.5021 (2)	0.15199 (11)	0.0479 (4)	
C11	0.98688 (13)	0.3641 (2)	0.16491 (13)	0.0465 (5)	
C12	1.07654 (12)	0.3852 (2)	0.23108 (12)	0.0434 (4)	
C13	1.09506 (12)	0.5358 (2)	0.28358 (11)	0.0413 (4)	
C14	0.95979 (13)	0.6770 (2)	0.18378 (12)	0.0430 (4)	
H14A	0.9057	0.7363	0.1961	0.052*	
C15	1.03617 (14)	0.8184 (2)	0.32473 (12)	0.0458 (4)	
H15A	1.0042	0.9165	0.2910	0.055*	
H15B	1.1011	0.8509	0.3480	0.055*	
N1'	0.99468 (11)	0.7833 (2)	0.39819 (10)	0.0448 (4)	
C2'	1.04278 (15)	0.7769 (2)	0.48300 (13)	0.0487 (5)	
C3'	0.97606 (16)	0.7351 (3)	0.53885 (14)	0.0597 (6)	
H3'A	0.9929	0.6260	0.5702	0.072*	
H3'B	0.9765	0.8268	0.5818	0.072*	
C4'	0.88213 (17)	0.7215 (4)	0.47546 (16)	0.0776 (7)	
H4'A	0.8403	0.8085	0.4891	0.093*	
H4'B	0.8556	0.6071	0.4794	0.093*	
C5'	0.89524 (14)	0.7509 (3)	0.38388 (14)	0.0592 (6)	
H5'A	0.8765	0.6489	0.3469	0.071*	
H5'B	0.8591	0.8504	0.3559	0.071*	
OW1	0.83056 (15)	0.0651 (3)	0.20076 (15)	0.0845 (6)	
HW1	0.863 (2)	0.115 (5)	0.171 (2)	0.117 (12)*	
HW2	0.8177 (19)	0.149 (4)	0.2346 (19)	0.090 (9)*	
OW2	0.79729 (15)	0.3018 (3)	0.31763 (13)	0.0821 (6)	
HW3	0.821 (2)	0.270 (4)	0.374 (2)	0.095 (9)*	
HW4	0.758 (2)	0.386 (4)	0.3106 (19)	0.096 (10)*	

### Atomic displacement parameters (Å^2^)

**Table:**

	*U*^11^	*U*^22^	*U*^33^	*U*^12^	*U*^13^	*U*^23^
O1	0.0633 (9)	0.0471 (8)	0.0879 (11)	−0.0075 (7)	0.0235 (8)	−0.0212 (8)
O2	0.0591 (9)	0.0927 (12)	0.0482 (8)	−0.0114 (8)	0.0051 (7)	−0.0047 (8)
C1	0.0625 (13)	0.0511 (12)	0.0629 (13)	0.0107 (10)	0.0261 (11)	−0.0026 (10)
C2	0.0616 (13)	0.0718 (15)	0.0672 (14)	0.0249 (12)	0.0202 (11)	0.0051 (12)
C3	0.0507 (11)	0.0801 (16)	0.0532 (12)	0.0116 (11)	0.0133 (9)	0.0056 (11)
C4	0.0540 (11)	0.0571 (12)	0.0461 (10)	−0.0001 (9)	0.0166 (9)	−0.0010 (9)
N5	0.0551 (9)	0.0424 (9)	0.0429 (8)	0.0073 (7)	0.0090 (7)	−0.0068 (7)
C6	0.0621 (12)	0.0437 (11)	0.0499 (11)	0.0016 (9)	0.0180 (9)	−0.0025 (9)
C7	0.0809 (15)	0.0666 (14)	0.0470 (11)	0.0201 (12)	0.0155 (11)	0.0014 (10)
C8	0.0633 (14)	0.0802 (16)	0.0533 (12)	0.0202 (12)	0.0005 (10)	−0.0174 (12)
C9	0.0429 (11)	0.0660 (14)	0.0793 (15)	0.0003 (10)	0.0036 (10)	−0.0189 (12)
N10	0.0432 (8)	0.0439 (9)	0.0561 (10)	−0.0027 (7)	0.0117 (7)	−0.0062 (7)
C11	0.0499 (11)	0.0399 (10)	0.0568 (11)	−0.0041 (8)	0.0262 (9)	−0.0043 (9)
C12	0.0471 (10)	0.0422 (10)	0.0468 (10)	0.0005 (8)	0.0228 (8)	0.0010 (8)
C13	0.0447 (10)	0.0433 (10)	0.0407 (9)	0.0009 (8)	0.0197 (8)	0.0039 (8)
C14	0.0462 (10)	0.0391 (10)	0.0451 (10)	0.0054 (8)	0.0143 (8)	−0.0067 (8)
C15	0.0566 (11)	0.0384 (10)	0.0437 (10)	−0.0016 (8)	0.0151 (8)	−0.0027 (8)
N1'	0.0491 (9)	0.0459 (9)	0.0403 (8)	−0.0004 (7)	0.0127 (7)	−0.0021 (7)
C2'	0.0602 (12)	0.0412 (10)	0.0449 (11)	0.0002 (9)	0.0137 (9)	−0.0056 (8)
C3'	0.0771 (15)	0.0569 (13)	0.0509 (11)	0.0040 (11)	0.0267 (11)	0.0016 (10)
C4'	0.0624 (14)	0.108 (2)	0.0688 (15)	0.0060 (14)	0.0275 (12)	0.0141 (14)
C5'	0.0500 (12)	0.0689 (14)	0.0590 (12)	−0.0016 (10)	0.0143 (10)	0.0054 (11)
OW1	0.1068 (15)	0.0582 (11)	0.1047 (15)	−0.0158 (10)	0.0570 (13)	−0.0134 (10)
OW2	0.0982 (14)	0.0904 (14)	0.0565 (11)	0.0277 (12)	0.0171 (10)	−0.0008 (10)

### Geometric parameters (Å, °)

**Table:**

O1—C11	1.241 (2)	C9—H9A	0.9700
O2—C2'	1.228 (2)	C9—H9B	0.9700
C1—C2	1.372 (3)	N10—C11	1.345 (2)
C1—C12	1.391 (3)	N10—C14	1.463 (2)
C1—H1A	0.9300	C11—C12	1.472 (3)
C2—C3	1.377 (3)	C12—C13	1.403 (3)
C2—H2A	0.9300	C14—H14A	0.9800
C3—C4	1.382 (3)	C15—N1'	1.454 (2)
C3—H3A	0.9300	C15—H15A	0.9700
C4—C13	1.394 (3)	C15—H15B	0.9700
C4—H4A	0.9300	N1'—C2'	1.337 (2)
N5—C13	1.380 (2)	N1'—C5'	1.453 (2)
N5—C15	1.445 (2)	C2'—C3'	1.505 (3)
N5—C14	1.448 (2)	C3'—C4'	1.494 (3)
C6—C14	1.519 (3)	C3'—H3'A	0.9700
C6—C7	1.526 (3)	C3'—H3'B	0.9700
C6—H6A	0.9700	C4'—C5'	1.505 (3)
C6—H6B	0.9700	C4'—H4'A	0.9700
C7—C8	1.512 (3)	C4'—H4'B	0.9700
C7—H7A	0.9700	C5'—H5'A	0.9700
C7—H7B	0.9700	C5'—H5'B	0.9700
C8—C9	1.513 (4)	OW1—HW1	0.84 (4)
C8—H8A	0.9700	OW1—HW2	0.88 (3)
C8—H8B	0.9700	OW2—HW3	0.90 (3)
C9—N10	1.462 (3)	OW2—HW4	0.86 (3)
			
C2—C1—C12	121.2 (2)	C1—C12—C13	119.86 (18)
C2—C1—H1A	119.4	C1—C12—C11	119.34 (18)
C12—C1—H1A	119.4	C13—C12—C11	120.69 (16)
C1—C2—C3	118.8 (2)	N5—C13—C4	123.00 (17)
C1—C2—H2A	120.6	N5—C13—C12	118.60 (16)
C3—C2—H2A	120.6	C4—C13—C12	118.38 (17)
C2—C3—C4	121.4 (2)	N5—C14—N10	111.47 (14)
C2—C3—H3A	119.3	N5—C14—C6	115.00 (16)
C4—C3—H3A	119.3	N10—C14—C6	109.06 (15)
C3—C4—C13	120.2 (2)	N5—C14—H14A	107.0
C3—C4—H4A	119.9	N10—C14—H14A	107.0
C13—C4—H4A	119.9	C6—C14—H14A	107.0
C13—N5—C15	122.74 (16)	N5—C15—N1'	112.75 (15)
C13—N5—C14	120.72 (15)	N5—C15—H15A	109.0
C15—N5—C14	116.34 (15)	N1'—C15—H15A	109.0
C14—C6—C7	109.68 (17)	N5—C15—H15B	109.0
C14—C6—H6A	109.7	N1'—C15—H15B	109.0
C7—C6—H6A	109.7	H15A—C15—H15B	107.8
C14—C6—H6B	109.7	C2'—N1'—C5'	114.44 (17)
C7—C6—H6B	109.7	C2'—N1'—C15	124.13 (16)
H6A—C6—H6B	108.2	C5'—N1'—C15	121.42 (15)
C8—C7—C6	111.61 (18)	O2—C2'—N1'	124.92 (19)
C8—C7—H7A	109.3	O2—C2'—C3'	126.64 (18)
C6—C7—H7A	109.3	N1'—C2'—C3'	108.44 (18)
C8—C7—H7B	109.3	C4'—C3'—C2'	105.55 (17)
C6—C7—H7B	109.3	C4'—C3'—H3'A	110.6
H7A—C7—H7B	108.0	C2'—C3'—H3'A	110.6
C7—C8—C9	111.73 (18)	C4'—C3'—H3'B	110.6
C7—C8—H8A	109.3	C2'—C3'—H3'B	110.6
C9—C8—H8A	109.3	H3'A—C3'—H3'B	108.8
C7—C8—H8B	109.3	C3'—C4'—C5'	107.34 (18)
C9—C8—H8B	109.3	C3'—C4'—H4'A	110.2
H8A—C8—H8B	107.9	C5'—C4'—H4'A	110.2
N10—C9—C8	110.01 (18)	C3'—C4'—H4'B	110.2
N10—C9—H9A	109.7	C5'—C4'—H4'B	110.2
C8—C9—H9A	109.7	H4'A—C4'—H4'B	108.5
N10—C9—H9B	109.7	N1'—C5'—C4'	104.21 (17)
C8—C9—H9B	109.7	N1'—C5'—H5'A	110.9
H9A—C9—H9B	108.2	C4'—C5'—H5'A	110.9
C11—N10—C9	120.38 (16)	N1'—C5'—H5'B	110.9
C11—N10—C14	122.58 (15)	C4'—C5'—H5'B	110.9
C9—N10—C14	112.77 (16)	H5'A—C5'—H5'B	108.9
O1—C11—N10	121.74 (18)	HW1—OW1—HW2	104 (3)
O1—C11—C12	121.68 (18)	HW3—OW2—HW4	114 (3)
N10—C11—C12	116.53 (16)		

### Hydrogen-bond geometry (Å, °)

**Table:**

*D*—H···*A*	*D*—H	H···*A*	*D*···*A*	*D*—H···*A*
Ow1—Hw1···O1	0.84 (4)	1.998 (37)	2.818 (3)	167 (3)
Ow1—Hw2···Ow2	0.88 (3)	1.828 (33)	2.703 (3)	172 (3)
Ow2—Hw4···Ow1^i^	0.86 (3)	1.875 (34)	2.733 (3)	177 (3)
Ow2—Hw3···O2^ii^	0.90 (3)	1.864 (32)	2.764 (3)	178 (3)

Symmetry codes:  (i) −*x*+3/2, *y*+1/2, −*z*+1/2; (ii) −*x*+2, −*y*+1, −*z*+1.
